# *Candida albicans* exploits N-acetylglucosamine as a gut signal to establish the balance between commensalism and pathogenesis

**DOI:** 10.1038/s41467-023-39284-w

**Published:** 2023-06-26

**Authors:** Dandan Yang, Mao Zhang, Chang Su, Bin Dong, Yang Lu

**Affiliations:** 1 Hubei Key Laboratory of Cell Homeostasis, College of Life Sciences, TaiKang Center for Life and Medical Sciences, Wuhan University, Wuhan, 430072 China; 2 Hubei Key Laboratory of Cell Homeostasis, College of Life Sciences, Wuhan University, Wuhan, 430072 China

**Keywords:** Fungal pathogenesis, Pathogens

## Abstract

*Candida albicans* is a benign member of gut microbiota, but also causes life-threatening disseminated infections, suggesting that this fungus commensalism has evolved with retention of virulence traits. Here we reveal that N-acetylglucosamine (GlcNAc) enables *C. albicans* to balance between commensalism and pathogenesis. Although GlcNAc catabolism is beneficial for commensal growth of *C. albicans*, deleting GlcNAc sensor-transducer Ngs1 confers enhanced fitness, indicating that GlcNAc signaling is detrimental to commensalism. Interestingly, addition of GlcNAc attenuates commensal fitness of gut-evolved *C. albicans* but retains its disease-causing potential. We further demonstrate that GlcNAc is a major inducer of hypha-associated transcription in the gut, which represents the key determinant for commensal-pathogenic equilibrium. In addition to yeast-to-hypha morphogenesis, we also identify other factors, including Sod5 and Ofi1, that contribute to the balance. Thus, *C. albicans* uses GlcNAc to build up a tradeoff between fungal programs supporting commensalism and virulence, which may explain its success as a commensal and pathogen.

## Abstract (web summary)

The mechanisms by which opportunistic pathogens shift between commensalism and pathogenesis remains poorly understood. Here, the authors show that N-acetylglucosamine, a carbon source enriched in the gut, plays a role in modulating commensal-pathogenic balance in *Candida albicans*.

## Introduction

The yeast *Candida albicans* is a component of the human orogastrointestinal and genital microbiota where it can exist in a commensal state without causing pathological infection. The fungus lives and coevolves in these complex environments of the human body. Nonetheless, it is the most common fungal pathogen of humans, responsible for diseases ranging from superficial infections of the skin and mucous membranes to highly morbid, invasive infections of internal organs^1–3^, which suggests that *C. albicans* commensalism has evolved with retention of fungal virulence traits. Among these, the transition from yeast to hyphal cell morphology is central to *C. albicans*’ pathogenic potential, as strains defective in hyphal formation are attenuated in causing invasive infections in mice^4–6^. In contrast to its crucial role in pathogenicity, the hypha-associated transcription was recently shown to inhibit commensal fitness^7^. In this situation, fungal programs supporting commensalism and virulence seem to antagonize each other. While most studies focused on the identification of fungal factors involved in commensalism and pathogenicity^7,8^, little is known about the host factor(s) that determine this antagonistic relationship.

N-acetylglucosamine (GlcNAc) is a major structural component on the cell surface of a wide range of cells^9^. It is a component of the peptidoglycan of bacterial cell walls and the extracellular matrix glycosaminoglycans of animal cells. Because mammals digest and absorb dietary starch and oligosaccharides in the proximal small bowel, glucose is relatively depleted more distally, while gut bacteria can release GlcNAc during cell wall recycling^10^. GlcNAc is proposed to be a major carbon source to support the growth of *C. albicans* in humans^11^. In addition to supporting growth as an energy source, GlcNAc is a powerful inducer of morphological transitions, including hyphal development and phenotypic switching^12,13^, which is linked to its virulence, infection of distinct host niches and immune evasion^14,15^. The GlcNAc-sensor and transducer Ngs1 is the master regulator of GlcNAc signaling. It controls GlcNAc-induced expression of genes for GlcNAc catabolism, uptake, scavenging and pathogenesis^16^. As the focus of recent research has been on understanding how GlcNAc promotes *C. albicans* pathogenicity^17^, we know little about the exact role of this sugar on the regulation of commensalism within the healthy human host.

Here, we report that disruption of *NGS1* conferred enhanced commensal fitness, which occurs independently of GlcNAc catabolism. Rather, Ngs1-mediated gene expression program including hypha-specific transcription, as well as other factors such as Ofi1, determines this hypercompetitive phenotype. By serially passaging *C. albicans* in gastrointestinal (GI) tract of mice, we showed that cells recovered from murine gut enriched in GlcNAc produced a commensal defect, but retained virulent attributes to some extent, compared to that recovered from the gut enriched in glucose or with no additional sugar. Thus, *C. albicans* has exploited GlcNAc as a GI-specific signal to balance between commensalism and virulence.

## Results

### Disrupting GlcNAc signaling in *C. albicans* confers enhanced commensal fitness

**Fig. 1:**
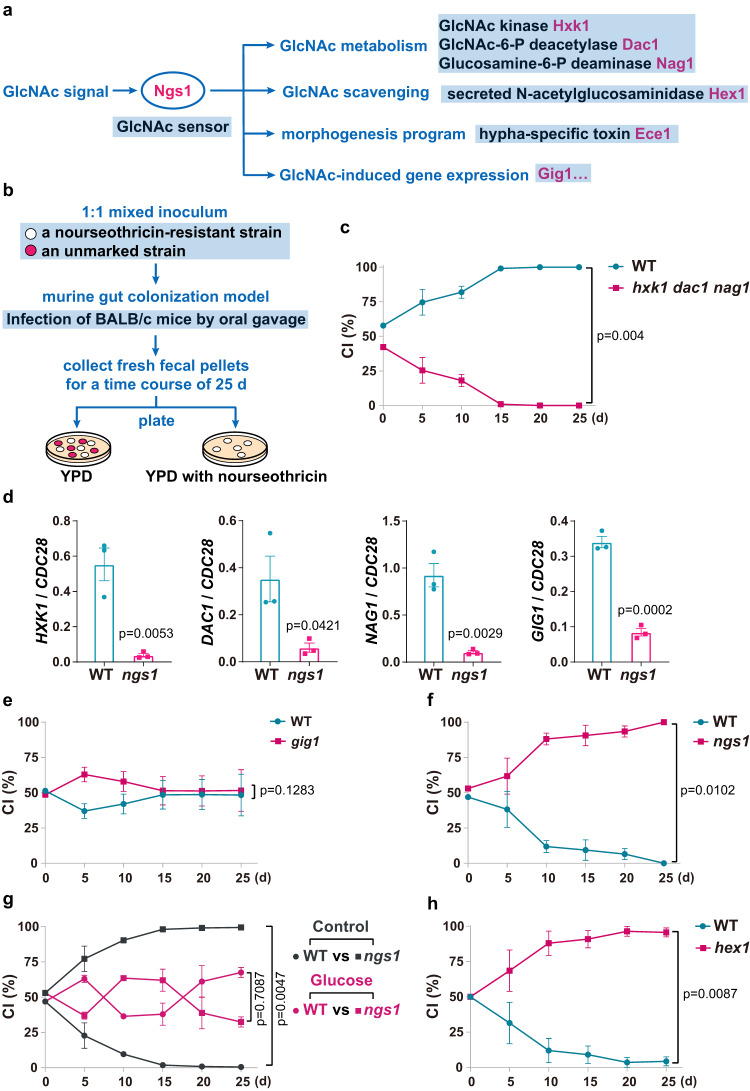
Deleting NGS1 that blocks GlcNAc signaling leads to enhanced commensal fitness. **a** Model of GlcNAc signaling in *C. albicans*. Binding of GlcNAc to Ngs1, the sensor and signal transducer of GlcNAc, controls the transcription of GlcNAc-responsive genes. **b** Schematic of commensal competition experiments. 6–8-week-old BALB/c mice were garaged with 1:1 mixtures of a nourseothricin-resistant strain and an unmarked strain. Relative abundance of strains was monitored over a time course of 25 days by collecting fresh fecal pellets and plating homogenates on YPD plates supplemented with or without 200 μg/ml nourseothricin. The competitive index (CI) was shown as the proportion of the indicated strain to the total. **c** Competition between wild type and *hxk1 dac1 nag1* triple mutant strain. *n* = 4 mice housed separately. Data are presented as the mean ± SEM. Significance was determined using the two-tailed paired Student’s *t* test. **d** qRT-PCR analysis of GlcNAc-induced genes in WT and *ngs1* mutant during commensal growth. BALB/c mice were infected by oral gavage with 10^8^ cells of indicated strain. After 3 days, RNA was extracted from luminal contents of large intestines. The expression levels of indicated genes in wild-type strain and *ngs1* mutant were determined by qRT-PCR analysis and showed as the means ± SEM. *n* = 3 biologically independent samples. Significance was determined using the unpaired two-tailed Student’s *t* test. The commensal fitness of *gig1* mutant (**e**) and *ngs1* mutant (**f**) was examined as described in **b**. **g** Competition between wild type and *ngs1* mutant strain in mice treated with or without 5% glucose. The CI was shown as the proportion of the tested strain to the total in the control group (indigo blue) and the glucose-treated group (magenta). **h** Competition between wild type and *hex1* mutant strain. **e**–**h** *n* = 3 mice housed separately. Data are presented as the mean ± SEM. Significance was determined using the two-tailed paired Student’s *t* test. Source data are provided as a Source Data file.

GlcNAc is an abundant carbon source supplied in the mammalian GI tract. It has been well established that GlcNAc catabolism is important for some bacteria to persist in mammalian gut^18^. To determine whether it is the case for the commensal-pathogenic fungus *C. albicans*, a competition experiment was conducted in which mice were orally inoculated with 1:1 mixtures of a nourseothricin-resistant (NAT^r^) WT strain and an unmarked strain in which genes encoding all three GlcNAc catabolic enzymes are deleted (*hxk1 dac1 nag1*) (Fig. 1a, b). NAT marker was shown to have no effect on commensal fitness^19^. At various times postinoculation, fecal pellets were collected from mice, homogenized, and plated to determine the number of CFU per gram of sample (CFU/g) and the competitive index (CI, the ratio of the indicated strain) (Fig. 1b). As expected, *hxk1 dac1 nag1* triple mutant exhibited reduced commensal fitness in competition with WT, suggesting that the ability to utilize GlcNAc as an energy source is also critical for gut commensal fitness of *C. albicans* (Fig. 1c). This result reinforces the opinion that GlcNAc metabolism is implicated in commensal growth of *C. albicans* in the GI tract^10^.

Ngs1 is the master regulator of GlcNAc-induced cellular programs including catabolism, scavenging, morphological transition and other processes (Fig. 1a). Consistent with its essential role for GlcNAc utilization under in vitro conditions, we detected a dramatic decrease in the expression of GlcNAc catabolic genes including *HXK1*, *DAC1* and *NAG1*, as well as *GIG1*, another GlcNAc-inducible gene^20^, in *ngs1* mutant compared to that in wild type during gut colonization (Fig. 1d), revealing that GlcNAc signaling must be encountered by *C. albicans* when it colonizes the gut. However, unlike the reduced fitness with *hxk1 dac1 nag1* triple mutant during commensal growth, *gig1* mutant and WT exhibit similar commensal fitness in a direct competition experiment (Fig. 1e). In addition to supporting growth as an energy source, GlcNAc often acts as a signaling molecule to induce multiple cellular programs via its sensor-transducer Ngs1 (Fig. 1a). We next asked, other than catabolism, whether GlcNAc signaling exerts influence on the fitness of *C. albicans* as a gut commensal. To this end, we compared the competitive fitness of WT and *ngs1* mutant directly in the same animals. In contrast to our expectation, the *NGS1* deletion mutant is hypercompetitive in the gut colonization model such that *ngs1* mutant outcompeted WT strain at time points as early as 10 days after inoculation (Fig. 1f). At day 25, all the WT cells seemed eliminated during colonization (Fig. 1f).

*WOR1*, a master regulator of commensalism in *C. albicans*^10^, was shown to be upregulated in the *ngs1* mutant from a global transcription profile in a GlcNAc-independent manner^21^. The upregulation of *WOR1* in *ngs1* mutant was confirmed by a qRT-PCR analysis in a murine colonization model (Supplementary Fig. 1a). To exclude the possibility that the enhanced commensal fitness of *ngs1* mutant is attributed to its increased *WOR1* expression, *WOR1* was deleted in the *ngs1* mutant. As shown in Supplementary Fig. 1b, *ngs1 wor1* double mutant exhibited hypercompetitive phenotypes over *wor1* single mutant, suggesting that the enhanced fitness in *ngs1* mutant cannot be solely due to the higher expression level of *WOR1*. Instead, the inability to respond to GlcNAc signaling in the gut appears to be key determinants for the hypercompetitative phenotype of the *ngs1* mutant. In support of this hypothesis, the fitness advantage of *ngs1* mutant was diminished when mice were fed with 5% glucose that blocked GlcNAc signaling in the gut (Fig. 1g). Additional support for this idea comes from data of the *hex1* mutant where disruption of *HEX1*, which encodes an N-acetylglucosaminidase responsible for external GlcNAc scavenging of *C. albicans* (Fig. 1a)^22^, conferred fitness advantage (Fig. 1h). Interestingly, *hex1* mutant even outcompeted *ngs1* mutant during gut colonization (Supplementary Fig. 1c). Taken together, these data suggest that GlcNAc-induced cellular programs play an inhibitory role towards *C. albicans* fitness when it colonizes the gut. This detrimental effect is dominant over the beneficial effect exerted by GlcNAc as energy source that promotes commensal growth.

### GlcNAc reshapes the gut evolution of *C. albicans*

**Fig. 2:**
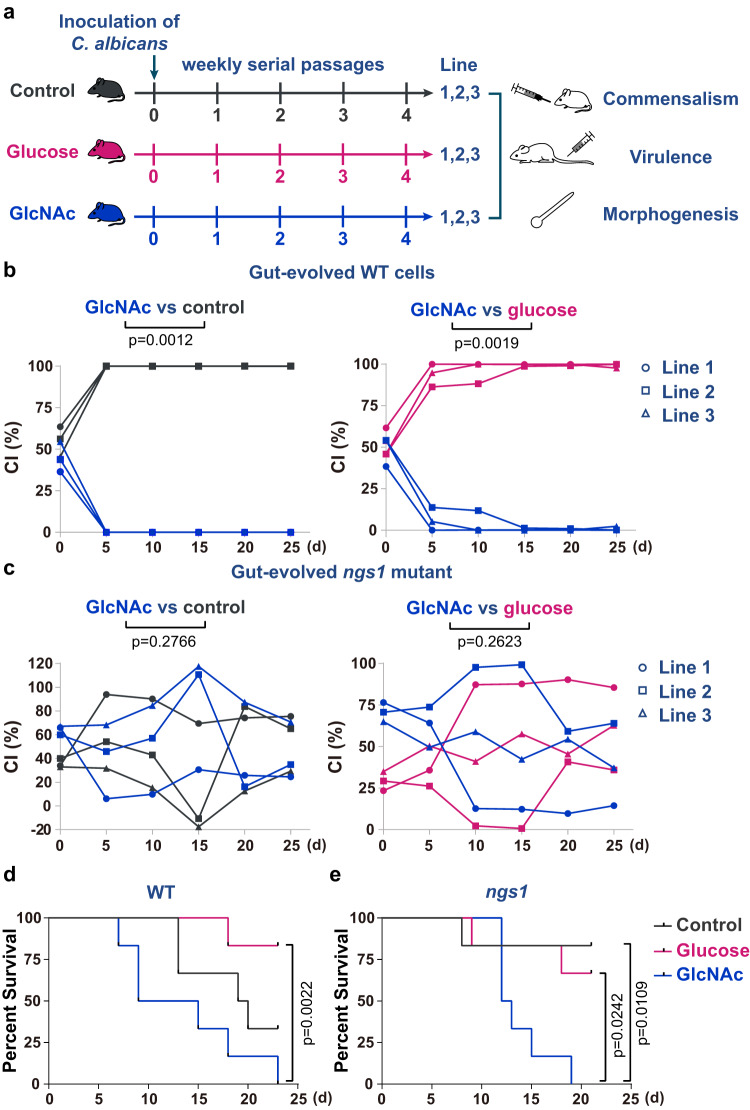
The virulent properties of gut-evolved C. albicans are retained to some extent upon GlcNAc adaptation. **a** Schematic overview of the evolution protocol based on the gut colonization of mice by *C. albicans* cells coupled with serial transplants from colonized to naïve hosts. Cells recovered from each evolution line were harvested after 4 weekly serial passages for the following experiments. The commensal fitness of gut-evolved wild-type (**b**) and *ngs1* mutant strain (**c**) in mice treated with different sugars (color coded) was determined as in Fig. 1b. Cells recovered from the three independent evolution line are indicated with different symbols. Left: control vs GlcNAc; right: glucose vs GlcNAc. Significance was measured with the one-tailed paired Student’s *t* test. *n* = 3 mice housed separately for each competitive fitness experiment. **d** GlcNAc signal outweighs the selection by the host to protect *C. albicans* from losing its virulence program. 18–21 g ICR mice were inoculated with 2.5 × 10^5^ of wild-type cells harvested after 4 weekly serial passages by tail vein injection. Significance was measured with log-rank test. *n* = 6 mice. **e** The survival curve of mice injected with *ngs1* mutant cells harvested from mice treated with different sugars after 4 weekly serial passages. Significance was measured with log-rank test. *n* = 6 mice. Source data are provided as a Source Data file.

*C. albicans* can be experimentally induced to increase its competitive fitness in the gut of antibiotic-treated mice by means of adaptive evolution^8,23^. To further confirm that GlcNAc can directly inhibit *C. albicans* commensalism, wild-type or *ngs1* mutant *C. albicans* cells were inoculated into mice of three groups fed with 5% GlcNAc, 5% glucose or only water respectively (Fig. 2a). For each group, this experimental evolution was performed independently in three mice (Line 1–3, Fig. 2a). After a week, the feces were transplanted from colonized to naïve mice. By four weekly serial passages, gut-evolved cells were harvested and recovered. Given that different clonal isolates from the same evolution line might display phenotype variations^23^, we collected all gut-evolved colonies that recovered from each mouse as a whole to represent an independent evolution line, and then subjected them for the following experiments. Recovered cells from each evolution line in GlcNAc were randomly paired with that in glucose or water, and were then orally inoculated with 1:1 mixtures to examine competitive fitness (Fig. 2a). As shown in Fig. 2b, wild-type cells recovered from mice fed with GlcNAc across all three evolution lines exhibited diminished fitness compared with cells recovered from mice fed with glucose or water. By contrast, no significant difference in commensal fitness was observed in *ngs1* mutant cells in the serial fecal transplantation experiment in mice fed with GlcNAc, glucose or water (Fig. 2c).

Exploiting the fact that gut-evolved strains exhibited increased fitness upon commensalism but decreased fitness upon blood-borne infection^23^, we propose that although GlcNAc exerts inhibitory effect on commensal fitness, it may help retain the virulence potential of *C. albicans*. Recovered cells from all three independent evolution lines (Line 1–3) under each evolved condition were mixed as 1:1:1 ratio for the subsequent systemic infection assay. As expected, wild-type cells that recovered from murine gut enriched in GlcNAc exhibited significantly more virulent phenotype than that recovered from glucose (Fig. 2d). Although we could not detect a statistical significant difference in virulence between strains recovered from GlcNAc and water, mice infected with the former strain died observable faster than that infected with the latter one (Fig. 2d). *ngs1* mutant recovered from glucose showed a similar level of virulence to that recovered from water (Fig. 2e). However, GlcNAc can still enhance the pathogenicity of *ngs1* mutant during gut evolution (Fig. 2e), suggesting that GlcNAc sensing is involved in the inhibitory effect of GlcNAc on commensalism but not necessarily on the retention of virulence traits.

To confirm the critical role of GlcNAc on the regulation of commensal-pathogenic equilibrium, an additional independent evolution experiment was performed. Consistent with the previous results (Fig. 2b), all three clonal isolates recovered after 4 weeks evolution in the gut enriched in GlcNAc were strongly outcompeted by the clonal isolates that evolved in glucose or water in the mouse GI colonization model (Supplementary Fig. 2a). As expected, clonal isolates recovered from mice fed with water or glucose exhibited hypercompetitive phenotypes over the original WT strain SN250 (Supplementary Fig. 2b). By contrast, the fitness advantage was lost when cells were evolved in the gut enriched in GlcNAc (Supplementary Fig. 2b). We also observed a significant loss of virulence in cells recovered from mice fed with water or glucose (Supplementary Fig. 2c). However, cells evolved in the gut enriched in GlcNAc displayed only a slight decrease in virulence compared to that of SN250 (Supplementary Fig. 2c), implying that the ability to cause disease was mostly retained by GlcNAc during gut colonization. Altogether, these results indicate that the evolutionary process of *C. albicans* during gut colonization can be reshaped by GlcNAc that attenuates commensal fitness advantage but retains virulence potential of gut-evolved cells.

### GlcNAc retains the potentiality of gut-evolved cells to form hyphae

**Fig. 3:**
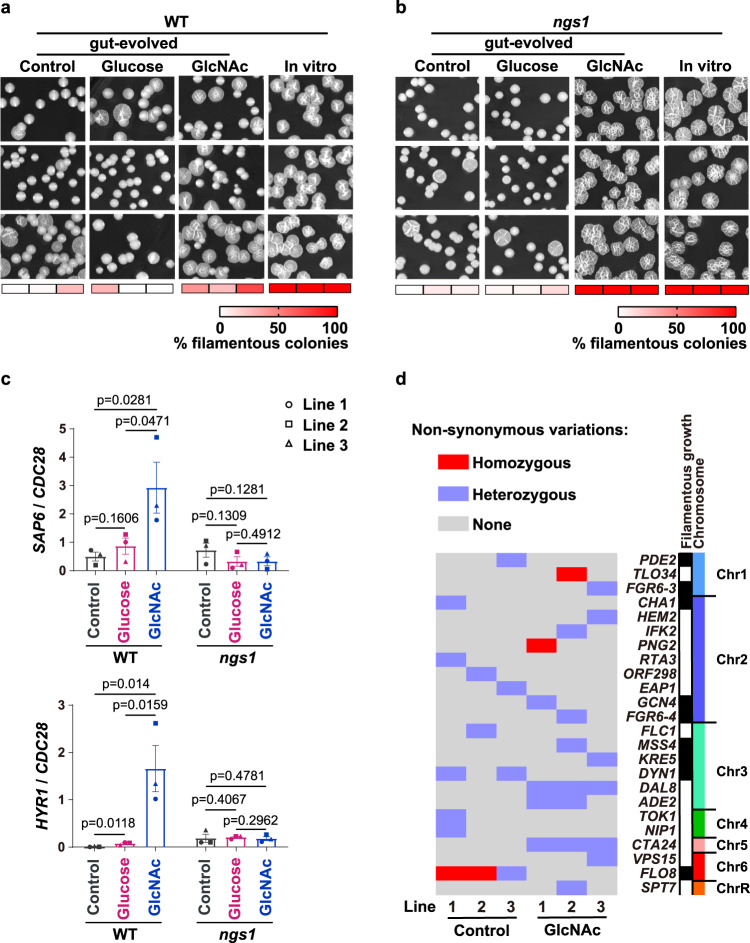
GlcNAc retains the ability of gut-evolved C. albicans to form hyphae. **a**, **b** Cells of wild type or *ngs1* mutant that collected from mice treated with indicated sugars after 4 weekly serial passages of three independent evolution experiment were incubated on Spider plates for hyphal induction. Photographs were taken after 3 days of growth at 37 °C. Cells evolved under in vitro conditions were served as control. **c** Expression levels of *SAP6* and *HYR1* in gut-evolved cells of WT and *ngs1* mutant during commensal growth, determined as described for Fig. 1d. Cells recovered from the three independent evolution lines are indicated with different symbols. Data are presented as the mean ± SEM of three independent evolution lines. Significance was determined using the one-tailed paired Student’s *t* test. **d** Nonsynonymous variations between gut-evolved *C. albicans* in GlcNAc and that in control water based on mutational pattern across 24 verified open-reading frames. Genes are ordered on the basis of chromosomal location. Source data are provided as a Source Data file.

Hypha-associated transcription in *C. albicans* inhibits commensalism but is crucial for its virulence^5,7^. This antagonistic regulation may contribute to the balance between commensal and pathogenic infection achieved by GlcNAc, as GlcNAc is a powerful inducer for hyphal morphogenesis. In agreement with a previous report^23^, *C. albicans* WT cells almost lost their ability to respond to hyphal-inducing stimuli after in vivo evolution (Fig. 3a). However, cells that serially passage through GI tract of mice fed with GlcNAc, but not glucose or water, were able to form hyphae in spider plates, although the ratio of wrinkled colonies was less than cells evolved under in vitro conditions (Fig. 3a). To our surprise, *ngs1* mutant cells after 4 weeks evolution in the gut enriched in GlcNAc retained the ability to form hyphae comparable to that evolved under in vitro conditions (Fig. 3b), although Ngs1 was shown essential for hyphal development in response to GlcNAc in vitro^16^. This may provide an explanation why *ngs1* mutant recovered from GlcNAc is much more virulent than that recovered from water or glucose (Fig. 2e). We next examined the expression of hypha-associated genes including *SAP6* and *HYR1*, both of which are involved in commensal fitness^7^, in gut-evolved cells. As expected, a dramatic increase in expression of *SAP6* and *HYR1* was detected in WT cells recovered from murine gut enriched in GlcNAc than that in glucose or water during gut colonization (Fig. 3c). Despite retaining a normal filamentation pattern in vitro (Fig. 3b), we could not detect any increase in the expression of *SAP6* or *HYR1* in *ngs1* mutant cells evolved in GlcNAc compared to that evolved in control water when colonized the gut (Fig. 3c). These results reinforce the opinion that cell morphology per se does not determine commensal fitness, but rather the hypha-associated transcription plays a critical role on it^7^.

In search of the genetic basis underlying GlcNAc-mediated retention of virulence of gut-evolved cells, one clonal isolate of WT strain was randomly picked from each independent evolution line (Line 1–3) in GlcNAc or control water for whole-genome sequencing. A total of 24 verified open-reading frames were found to contain ≥ 1 amino acid variation between gut-evolved strains adapted in GlcNAc and control water (Fig. 3d, Supplementary Data 1). On the basis of Gene Ontology (GO) enrichment analysis, 9 of these genes are related to filamentous growth (Fig. 3d). All three sequenced strains evolved in control water harbored loss-of-function mutations (premature stop codons or frame shifts) of *FLO8* (Fig. 3d), which encodes an essential transcription factor for hyphal morphogenesis^24^. This is in agreement with a previous report^23^. Notably, no mutation in *FLO8* was found in strains evolved in the gut enriched in GlcNAc compared to the reference strain SC5314 (Fig. 3d). Thus, we suggest that GlcNAc retains the ability of gut-evolved *C. albicans* to form hyphae through blocking mutations in *FLO8*, although variations in other genes may also contribute to GlcNAc-mediated retention of the capacity to form hyphae.

### GlcNAc is critical for hyphal morphogenesis program of *C. albicans* in the gut

**Fig. 4:**
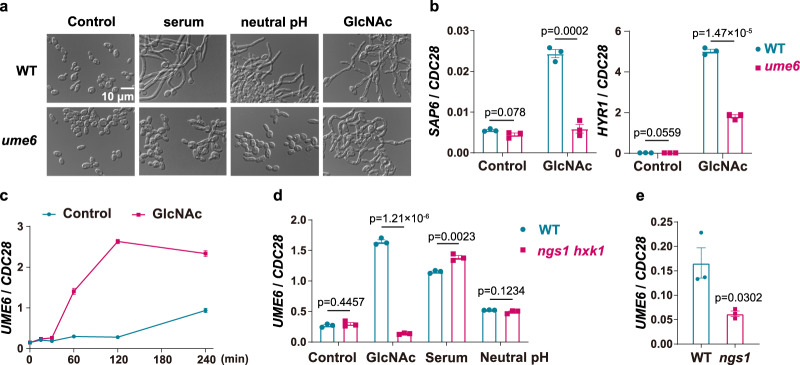
GlcNAc is critical for the normal expression of UME6 in the gut. **a** *ume6* mutant could form long filaments in response to GlcNAc. Overnight cultures of wild type and *ume6* mutant were diluted at 1:100 fold into SC galactose medium at 30 °C and incubated for 4 h. Cells were then treated with 10% serum, neutral pH or 50 mM GlcNAc for hyphal induction. The medium was buffered to pH 7 with HEPES. Photographs were taken after 3.5 h of incubation at 37 °C. Representative images of three biologically independent experiments are shown. Scale bar, 10 μm. **b** GlcNAc activates the expression of *SAP6* and *HYR1* in a Ume6-dependent manner. Overnight cultures of wild type and *ume6* mutant were diluted at 1:100 fold into SC galactose medium and incubated at 30 °C for 4 h. Log-phase cells were then treated with or without 50 mM GlcNAc at 37 °C. Samples were collected at 3.5 h upon GlcNAc treatment for qRT-PCR analysis. **c** *UME6* mRNA levels were determined by qRT-PCR analysis over a time course of GlcNAc-induction. WT cells in SC galactose medium were treated with or without 50 mM GlcNAc. Cells were collected after incubation for 0 min, 15 min, 30 min, 60 min, 2 h and 4 h at 37 °C. **d** Deletion of *NGS1* specifically abolished GlcNAc-responsive induction of *UME6*. Log phase cells were incubated as described in **b** and treated with GlcNAc or serum. SC galactose medium was buffered to pH 7 with HEPES. Cells were collected after 3.5 h of growth at 37 °C for RNA extraction. The expression level of *UME6* was quantified by qRT-PCR. **e** RNA extraction was performed as described in Fig. 1b. The expression levels of *UME6* in wild-type strain and *ngs1* mutant were determined by qRT-PCR analysis. **b**–**e** The signals obtained from *CDC28* mRNA were used for normalization in qRT-PCR analysis. Data are presented as the mean ± SEM. *n* = 3 biologically independent samples. Significance was measured with the two-tailed unpaired *t* test in GraphPad Prism. Source data are provided as a Source Data file.

A recent study reported that *ume6* null mutants undergo normal morphogenesis in the GI tract^7^, although they show filamentation defects under in vitro conditions^25^. It is well established that Ume6 functions as the terminal transcriptional activator of the hyphal gene regulatory network whose expression is highly induced during yeast-to-hyphae transition^25,26^. Interestingly, we found that GlcNAc, but not serum or neutral pH, could induce log-phase cells of *ume6* mutant to form normal hyphae as wild-type cells (Fig. 4a). However, *UME6* was necessary for the induction of hypha-associated proteins in response to GlcNAc, including Sap6 and Hyr1 (Fig. 4b). This is consistent with the notion that GlcNAc-induced expression of hypha-associated genes requires the presence of *UME6* that restrains the capacity of *C. albicans* for gut colonization^7^. Because GlcNAc was shown to inhibit the growth of the *ngs1* mutant at 37 °C in SD medium and the inhibitory effect could be removed by *HXK1* deletion^16^, the *ngs1hxk1* double mutant was used to examine the function of Ngs1 in hypha-associated *UME6* expression. *UME6* was dramatically upregulated upon GlcNAc induction in a Ngs1-dependent manner whereas Ngs1 is not required for *UME6* induction in response to serum or neutral pH (Fig. 4c, d). Remarkably, a significant lower level in *UME6* expression was observed in *ngs1* mutant than that in wild type strain after three days of colonization (Fig. 4e) and overexpression of *UME6* in *ngs1* produced a strong loss-of-fitness phenotype (Supplementary Fig. 3), suggesting that decreased expression of *UME6* contributes the enhanced fitness of *ngs1*. Given that Ngs1 is specifically required for hypha-specific transcription in response to GlcNAc^16^, the results, summarized in Fig. 3, clearly demonstrate that although the mammalian digestive tract is replete with signals that trigger yeast-to-hypha morphogenesis, GlcNAc plays a key role on hypha-associated program during gut colonization.

### GlcNAc-induced transcriptome regarding colonization and virulence

To further investigate the function of GlcNAc in the balance between commensalism and virulence, we performed independent transcriptome sequencing (mRNA-Seq) analyses to profile *ngs1* mutant and reference strain SN250. Animals were colonized with WT or *ngs1* (*n* = 3 mice/strain) for 3 days, and RNA was recovered from large intestine. For comparison, both strains were also propagated in vitro in the presence of GlcNAc or glucose at 37 °C for 0.5 h (*n* = 3 cultures, Fig. 4a). For each sample, a minimum of 25 million mRNA-Seq reads mapped to the *C. albicans* genome. We detected expression of 6281*C. albicans* genes or roughly 98% of the predicted genome under at least one experimental condition (Supplementary Data 2).

**Fig. 5:**
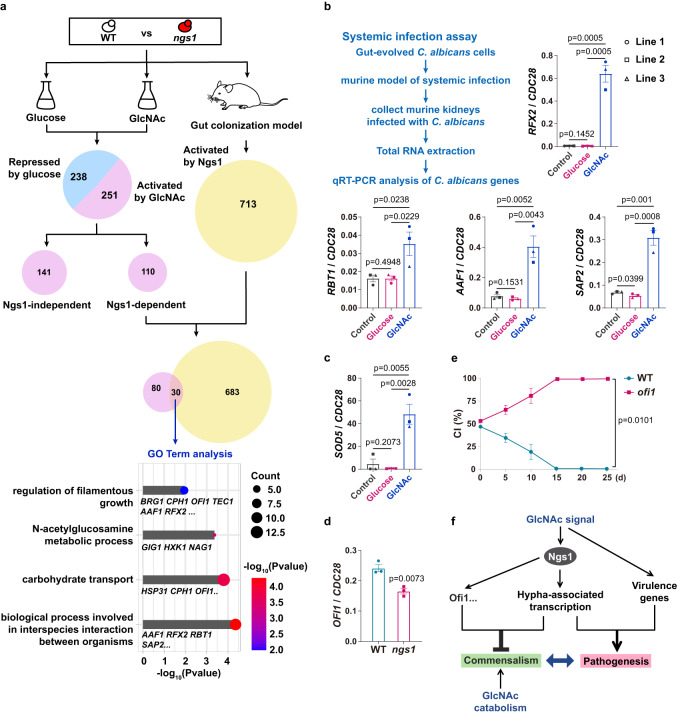
mRNA-Seq analysis reveals GlcNAc-mediated regulation of symbiosis and virulence. **a** Schematic of mRNA-Seq experiment. mRNA was recovered from WT (SN250) and *ngs1* propagated under in vitro conditions (glucose or GlcNAc, 37 °C, *n* = 3 cultures) or for 3 days in the GI commensalism model (*n* = 3 mice). Differentially expressed genes were defined by fold change ≥1.5 and a *P* value of <0.05 found by DESeq2 (1.34.0 in Rstudio). No adjustment for multiple comparisons was made. Venn diagram showing the overlap of Ngs1-dependent GlcNAc-activated genes between in vitro and in vivo murine gut conditions. Gene Ontology biological process enrichment analysis of the overlap genes is shown in the bottom. **b** Gut-evolved cells were harvested from mice treated with different sugars after 4 weekly serial passages. The resulting cells were subjected to 6–8-week-old BALB/c mice by tail vein injection. Total RNA was extracted from the kidneys after three days of infection. The mRNA levels of the indicated genes were determined by qRT-PCR and normalized with *CDC28*. **c** The expression level of *SOD5* was examined as described in **b**. **d** The mRNA level of *OFI1* from the intestinal contents of mice at the third day after gavage was assessed by qRT-PCR. Data are presented as the mean ± SEM. *n* = 3 biologically independent samples. Significance was determined using the two-tailed unpaired Student’s *t* test. **e** Competition between wild type and *ofi1* mutant strain. *n* = 3 mice housed separately. Data are presented as the mean ± SEM. **f** GlcNAc signal in the gut enables *C. albicans* to establish a balance between commensalism and pathogenicity. GlcNAc signaling controls hyphal morphogenesis in the gut via its sensor-transducer Ngs1, which is proposed to contribute primarily to this balance. Additional Ngs1-independent pathways may also play a role on this process. **b**, **c** Cells recovered from the three independent evolution lines are indicated with different symbols. Data are presented as the mean ± SEM of three independent evolution lines. Significance was determined using the one-tailed unpaired Student’s *t* test. Source data are provided as a Source Data file.

To identify genes that require Ngs1 for normal expression in the gut, we compare the transcriptomes of commensally propagated WT and *ngs1* strains. Using a 1.5-fold cutoff, there are 713 genes downregulated in *ngs1* compared to WT in the gut (Fig. 5a). We next asked whether these genes are activated by GlcNAc in vitro. 489 genes upregulated in wild-type cells by 1.5-fold or more upon GlcNAc induction compared to that in glucose (Fig. 5a). We further aimed at discriminating genes activated by GlcNAc from those repressed by glucose. All of the genes that were identified as upregulated in maltose compared to glucose were therefore removed from this setting^27^. This filter criteria led to a list of 251 genes as GlcNAc-activated genes, among which 110 genes are regulated by Ngs1 (Fig. 5a). There was a significant overlap of this gene cluster with previous defined gene set that activated by Ngs1 in the gut (*p* = 1.05 × 10^−26^, hypergeometric test). We then focused on these 30 genes and subjected them to function analysis using Gene Ontology (GO). As shown in Fig. 5a (bottom), they were significantly enriched in genes for GlcNAc metabolic process (*p* = 0.0004), filamentous growth (*p* = 0.01), carbohydrate transport (*p* = 0.0002) and interspecies interaction (*p* = 4.38 × 10^−5^). Based on the information on Candida Genome Database (CGD), some genes are shown to implicate in *C. albicans* virulence, among which we picked out four representative genes including *RBT1*, *SAP2*, *AAF1* and *RFX2*. Remarkably, they were dramatically induced in cells recovered from murine gut enriched in GlcNAc than that in glucose or water when they infected murine kidneys (Fig. 5b), supporting the capability of GlcNAc in virulence retention. We noticed that some of GlcNAc-responsive genes under in vitro conditions whose induction is independent of Ngs1 are clearly involved in pathogenicity of *C. albicans*, for example *SOD5*^28^. As expected, we detected an increase in the expression of *SOD5* in GlcNAc-evolved cells during invasive infection (Fig. 5c), indicating that GlcNAc retains *C. albicans* virulence potential also through Ngs1-independent pathways.

In addition to genes implicated in virulence, we noticed *OFI1* whose expression was induced by GlcNAc in a Ngs1-dependent manner both in vitro and in murine gut (Fig. 5a). *OFI1* was previously shown to promote phenotypic switching required for *C. albicans* mating^29^. The reduced expression of *OFI1* in *ngs1* mutant during gut colonization was confirmed by qRT-PCR (Fig. 5d). Given that the sexual switch is related to commensalism^30^, we asked whether decreased expression of *OFI1* contributes to the enhanced commensal fitness of *ngs1* mutant. To test this hypothesis, we profiled *ofi1* null mutant in the GI colonization model. As shown in Fig. 5e, *ofi1* mutant strongly outcompeted wild type in the mouse GI colonization model. Because deletion of *OFI1* has no effect on filaments formation at 37 °C (Supplementary Fig. 4), our data suggest that, in addition to hypha-associated transcriptome, other cellular program(s) might also contribute to the inhibitory effect of GlcNAc on commensal fitness.

## Discussion

*C. albicans* is the most prominent fungus residing in the human gut^31^, but also remains a deadly pathogen and a leading cause of bloodstream infections worldwide^1,32^. In order to develop successful approaches against candidiasis, it is necessary to understand how the balance between commensalism and virulence is established in the context of the complex host environment. In this study, we demonstrate that GlcNAc, an abundant carbon source in the GI tract, enables *C. albicans* to balance between commensalism and virulence (Fig. 5f). It represents a major inducer for the expression of hypha-associated genes, as well as other transcription programs in the mammalian gut (Fig. 5f), which allows *C. albicans* commensalism evolved with retention of virulence traits. Our work provides new insights into how a host-associated factor regulates the lifestyle of a commensal-pathogenic fungus.

GlcNAc is thought to be the primary carbon source of *C. albicans*, as it is used by the fungus as a signal for nutrient availability^11^. The ability to utilize GlcNAc as energy source is important for some bacteria to persist in mammalian gut^18^. We demonstrate that this is also the case for *C. albicans*. Ngs1 is the master regulator of GlcNAc signaling. To our surprise, deleting *NGS1*, confers a fitness advantage over wild-type strain (Fig. 1f), although the *ngs1* mutant is unable to catabolize GlcNAc^16^, indicating that GlcNAc signaling exerts inhibitory effect on commensalism, which is dominant over its beneficial influence as a carbon source to support commensal growth. As such, Ngs1 functions as an inhibitor of commensalism, although it is essential for GlcNAc catabolism. Through serial passage in the GI tract of antibiotic-treated mice, *C. albicans* adapted to be hyper-fit as a gut commensal but decreased fitness upon blood-borne infection. These gut-evolved variants display defects in hyphae formation^23^. Using a similar experimental evolution approach, we showed that addition of GlcNAc in the gut could reshape this evolution process, in which the fitness advantage of gut-evolved strains was attenuated, but the virulence traits, such as the ability to form hyphae, were retained by blocking mutations in *FLO8* (Figs. 2 and 3d). This indicates that although GlcNAc is disadvantageous for commensalism, it represents an evolutionary pressure to maintain key pathogenic attributes of *C. albicans* such that this commensal can cause diseases if host or environmental factors are permissive.

It is well documented that *C. albicans* morphogenesis program is central to the balance between gut commensalism and invasive infection. Witchley et al., reported that hyphal formation was observed throughout the gut, with greater abundance of hyphae over yeast cells in distal intestinal regions^7^. Although mammalian GI tract is replete with hypha-inducing signals, several lines of evidence presented in this study and literature suggest that GlcNAc serves as a major inducer for hyphal transcriptional program in *C. albicans* during gut colonization, thus dictating commensal-pathogenic properties of this fungus. First, GlcNAc, but not serum or neutral pH, could induce log phase cells of *ume6* mutant to form normal hyphae (Fig. 4a), indicating that GlcNAc should be a host-associated signal that accounts for the in vitro*-*in vivo discrepancy observed in the filamentation potential of *ume6* mutant. Second, despite normal filamentation observed in *ume6* mutant in GlcNAc, GlcNAc failed to induce the expression of some hypha-associated proteins in this strain, including *SAP6* and *HYR1*, both of which limit commensal fitness (Fig. 4b). This might lead to hyper-fit phenotype of *ume6* mutant. Third, Brg1, a key regulator in GlcNAc-induced filamentation of log phase cells^33^, is also critical for hyphal morphogenesis in the gut^7^. Fourth and most strikingly, deletion of *NGS1* results in a reduced *UME6* expression when *C. albicans* colonizes the gut (Fig. 4e). This finding underscores the exclusive role of GlcNAc on the regulation of hypha-associated transcription during gut colonization as Ngs1 is specifically required for morphological program in response to GlcNAc^16^.

As a natural inhabitant of the human GI tract, *C. albicans* lives and coevolves in this complex niche. Why GlcNAc stands out from numerous known factors that trigger yeast-to-hypha morphogenesis in the gut? This could be attributed to its exceptional ability to induce hyphal formation under conditions without inoculation^16,33^. While many environmental cues favor hyphal growth when saturated cells are diluted into fresh medium at 37 °C, only GlcNAc, serum and neutral pH were identified to date to trigger hyphal development in log phase cells^33,34^. Given that serum is usually absent from mammalian GI tract where pH is fluctuated, GlcNAc, an abundant sugar in this niche, should be a suitable gut signaling molecule to control hypha-associated program in *C. albicans* during colonization. In addition to morphological program, other factors regulated by Ngs1, such as Ofi1, might also contribute to attenuated fitness induced by GlcNAc. It was recently reported that *C. albicans* specifically modulates human Th17 responses, leading to heterologous immunity and protecting against other invading pathogens^35–37^. Commensal Th17 immunity primed by *C. albicans* is regulated via oscillating *UME6* expression^38^ and relies on the peptide toxin Candidalysin^35,39^. Given the fact that GlcNAc is a major inducer of hypha-associated transcription in the gut, we suggest that GlcNAc may represent a major evolutionary driving force for *C. albicans* to trigger systemic Th17 responses. Another more recent study revealed that a subset of immune cells detects *C. albicans* in the GI tract and leads to production of antifungal immunoglobulin G (IgG)^40^. GlcNAc-mediated expression of virulence traits may also contribute to this process. It will be interesting to test directly whether GlcNAc is important for this pathobiont to build up immunity against other pathogens.

## Methods

### Ethics statement

All animal experiments were approved by the Institutional Animal Care and Use Committee (IACUC) at Wuhan University and performed as outlined in the guide for the care and use of laboratory animals issued by the Ministry of Science and Technology of the People’s Republic of China.

### Media and growth conditions

*C. albicans* strains were routinely grown at 30 °C in YPD (2% Bacto peptone, 2% glucose, 1% yeast extract). Transformants were selected on SC medium (0.17% Difco yeast nitrogen base w/o ammonium sulfate, 0.5% ammonium sulfate and auxotrophic supplements) with 2% glucose or on YPD plate supplemented with 200 µg/ml nourseothricin.

Spider medium (1% mannitol, 1% nutrient broth, 0.2% K_2_HPO_4_) with 2% agar was used for colony morphology assay at 37 °C. Hyphal induction in liquid medium was conducted as described previously^16^. Briefly, *C. albicans* cells grown overnight at 30 °C in liquid YPD were washed three times with PBS, resuspended in an equal volume of PBS, and diluted 1:100 into liquid SC medium containing 50 mM galactose. After 4 h of incubation at 30 °C, hyphal growth was induced by a shift in temperature to 37 °C in combination with 50 mM GlcNAc. In addition to GlcNAc, 10% Serum and neutral pH were used for morphology assay in this study as well. For neutral pH condition, cells were pelleted and resuspended in SC galactose medium buffered to pH 7 with 15 mM HEPES buffer (pH 7). Cell morphology was detected using differential interference contrast (DIC) optics at 3.5 h after transferring to 37 °C.

### Plasmid and strain construction

The *C. albicans* strains used in this study are listed in Supplementary Table 1. Primers are listed in Supplementary Data 3. SC5314 genomic DNA was used as the template for all PCR amplifications of *C. albicans* genes.

The *hxk1 nag1 dac1* triple mutant strain was constructed by two-step homologous recombination in BWP17^41^, and two copies of the genes were replaced by *HIS1* and *ARG4*, respectively. *HIS1* and *ARG4* fragments are amplified from pGEM-HIS1 and pRS-ARG4ΔSpeI by primers 1 and 2. All deletions were confirmed by PCR.

Deletion of *GIG1* in *C. albicans* was conducted using *SAT1*-flipping strategy^42^. Upstream (primers 9 and 10) and downstream (primers 11 and 12) sequences of *GIG1* were cloned as ApaI-XhoI and NotI-SacII fragments, respectively, on both sides of the *SAT1* flipper cassette to obtain the plasmid pSFS2-GIG1. To disrupt the endogenous copies of *GIG1* in wild-type cells, pSFS2-GIG1 was linearized using KpnI and SacII, which was followed by two sequential rounds of transformation, selection, and recycling of the *SAT1* marker. All deletions were confirmed by PCR.

*WOR1* deletion was performed using CRISPR-Cas9 strategy as follows. The single guide RNA (sgRNA) (primers 3 and 4) was annealed to insert into the pV1093 vector^43^. The resulting plasmid was linearized by digestion with KpnI and SacI and transformed into WT or the *ngs1* mutant with the repair template (primers 5 and 6). The mutants were verified by sequencing.

The pBES116-SAT1 plasmid was constructed by amplifying the *SAT1* cassette (primers 7 and 8) from pSFS2 plasmid^42^. The PCR product was inserted into the NdeI-PstI site of pBES116 to create pBES116-SAT1 plasmid for strain construction in commensal competition experiments.

The pBES116-TDH3p-UME6 plasmid was constructed by amplifying *UME6* coding region with primers 13 and 14. The resulting PCR product was inserted into pBES116-TDH3p plasmid^33^.

### Commensal competition experiments in mouse

All animals were singly or doubly housed depending on experiment and provided autoclaved distilled water and autoclaved mouse chow. 6–8-week-old female BALB/c mice (purchased from Beijing Vital River Laboratory Animal Technology Company) were treated with antibiotics water (streptomycin, 2 mg/ml; penicillin, 0.97 mg/ml) for 3 days and then inoculated with a 1:1 mixture of a nourseothricin-resistant strain and an unmarked strain at 5 × 10^8^ cells/ml by oral gavage as previously reported^19^. The antibiotics water was used throughout the commensal competition experiment. Colonization was tested over time by collecting fresh fecal pellets and plating homogenates on YPD plates containing streptomycin (100 µg/ml) and ampicillin (50 µg/ml) supplemented with or without 200 μg/ml nourseothricin. The competitive index (CI) of the competition experiment has been shown as the proportion of the indicated strain to the total. Mice were housed in a temperature-constant animal room (22 °C) with reversed dark/light cycle (7:00 a.m. on and 7:00 p.m. off) and 40–70% humidity.

### Murine model of systemic infection

18–21 g male ICR mice were inoculated with 2.5 × 10^5^ *C. albicans* cells by tail vein injection. Mice were monitored daily for overall health condition. The survival curve was calculated using GraphPad Prism. To determine the expression of virulence factors in *C. albicans* during systemic infection, 6–8-week-old BABL/c male mice were inoculated with 2 × 10^6^ *C. albicans* cells by tail vein injection and infected kidneys were taken at the third day after injection for RNA extraction. Mice were housed in a temperature-constant animal room (22 °C) with reversed dark/light cycle (7:00 a.m. on and 7:00 p.m. off) and 40–70% humidity.

### In vivo gastrointestinal evolution

The evolution experiment was performed in 6–8-week-old female BALB/c mice in the presence of antibiotics as described previously with modifications^23^. Mice were first colonized with 10^8^ CFUs *C. albicans* cells. To minimize microbial transfer among cage mates, only one animal was housed per cage. After 7 days, a new set of naïve mice, pre-treated with antibiotics, was cohoused with the previous passage for 3 days. This treatment results in the successful colonization of *C. albicans* cells in the new set of mice. After 7 days, the procedure was repeated using another set of naïve mice as the recipients and serial passaging was performed weekly for 4 passages. At the end, animals were euthanized, and the content in large intestine was collected. Homogenates were grown on Sabouraud agar plates containing streptomycin (100 µg/ml) and ampicillin (50 µg/ml) for 24 h. All colonies were washed by PBS for subsequent experiments.

### RNA sequencing and analysis

6–8-week-old female BALB/c mice were colonized with a single *C. albicans* strain (WT SN250 or *ngs1* mutant). Three animals were colonized with each strain for 3 days and housed individually. Animals were euthanized, and the content in large intestine was flash frozen in liquid nitrogen and stored in −80 °C. For in vitro assays, *C. albicans* cells were grown in YPD medium to log phase at 30 °C, washed with PBS for three times, and then resuspended in YEP medium supplemented with 50 mM glucose or GlcNAc. Samples were collected after incubation at 37 °C for 0.5 h for RNA-seq analysis. Library preparation and sequencing of RNA was performed at Novogene (Beijing, China). A sequencing library was constructed by using a NEBNext Ultra RNA library prep kit for Illumina (New England BioLabs, USA). The RNA-Seq library was assessed by the Agilent Bioanalyzer 2100 system and quantified by qRT-PCR before sequencing on the Illumina NovaSeq platform. Clean reads were mapped to the *C. albicans* reference genome (SC5314_A21), and mapped reads were counted. Differentially expressed genes were defined by fold change ≥1.5 and a *P* value of <0.05 found by DESeq2 (1.34.0 in Rstudio). GO term process analysis was performed on the Candida Genome Database (http://www.candidagenome.org/cgi-bin/GO/goTermFinder) and viewed by ggplot2 (3.4.1).

### RNA extraction and quantitative PCR expression analysis

Total RNA from *C. albicans* cells incubated under the in vitro conditions was purified using the RNAprep pure Tissue Kit and DNase-treated at room temperature for 15 min using the RNase-free DNase Set (Tiangen). Total RNA in the contents from the large intestines of mice was extracted using the Fecal RNA Extraction Kit (Biotech). Additionally, total RNA in the infected kidney of mouse was extracted using RNAiso Plus (TaKaRa). cDNA was synthesized using the Maxima^TM^ H Minus cDNA Synthesis Master Mix with dsDNase (Thermo), and qPCR was done using the iQ SYBR Green Supermix (Bio-Rad). The primers for qRT-PCR were listed in Supplementary Data 3. The signals obtained from *CDC28* mRNA were used for normalization. All data showed the average of three independent qRT-PCR experiments with error bars representing the SEM.

### Whole-genome sequencing of *C. albicans* strains

Genomic DNA was extracted using plant genome DNA extraction kit (Sangon, Shanghai, China). The library preparation and next generation sequence was performed by Sangon Biotech (Shanghai) Co., Ltd. 500 ng quantified DNA was randomly fragmented by Covaris (Woburn, USA). Whole-genome sequencing libraries were prepared using Illumina Hieff NGS MaxUp II DNA Library Prep Kit (YEASEN, Shanghai, China). The library concentration and size was confirmed by Qubit 4.0 (Thermo, Waltham, USA) and 2% agarose gel electrophoresis respectively. Then the libraries were pooled and loaded on Novaseq 6000 (Illumina, San Diego, USA)/DNBseq-T7 (BGI, Shenzhen, China) to generate indexed paired-end reads of 2 × 150 bp.

Raw reads containing adaptor sequences and those with ambiguous or low-quality bases at the beginning or end were trimmed using Fastp. The qualified reads from each sample were aligned to the assembled *C. albicans* SC5314 reference genome (http://www.candidagenome.org/download/sequence/C_albicans_SC5314/Assembly21/current/) using BWA (0.7.17) with default parameters. Duplicated reads were removed and coverage values were calculated using SAMTOOLS. Primary variation calling was conducted using the Genome Analysis ToolKit (GATK, v4.1.2). The filtering of low-quality variants using the criteria “QD <2.0||MQ <40.0||FS> 60.0 ||| ReadPosRankSum <−8.0 || MQRankSum <−12.5||SOR> 3.0” generated the final variant set. The bam file produced from the mapping procedure was analyzed for structural variations (SVs) detection by DELLY (0.8.1) with default parameters. Copy number variations (CNVs) were detected with CALL in CNVnator (0.4). Functional annotation of all the genetic variants was completed by snpEff (4.3t). Comparing the different non-synonymous variations of control group with that of GlcNAc group, genes including specific variations occurring in either group were presented. GO term process analysis of the non-synonymous effect-coding genes was performed on the Candida Genome Database and *p*-values < 0.05 was considered as significantly enriched.

### Statistical analysis

All experiments were performed with at least three biological repeats except indicated in the figure legends, and no statistical method was used to predetermine sample sizes. Analyses were conducted using Graphpad Prism 9.0 software. Results are expressed as the mean ± SEM as indicated, and analyzed using Student’s *t* test. *P* values of less than 0.05 were considered statistically significant.

### Reporting summary

Further information on research design is available in the Nature Portfolio Reporting Summary linked to this article.

## Supplementary information

Supplementary Information

Description of Additional Supplementary Files

Data 1

Data 2

Data 3

Reporting Summary

## Data Availability

RNA-Seq data and genome sequencing data that support the findings of this study have been deposited in the Genome Sequence Archive (GSA) under the accession code CRA010506 and CRA010507 respectively. Reference genomes and genome annotations were obtained from Candida Genome Database. The generated strains in this study are available from the corresponding author upon request. Source data are provided with this paper.

## Source data

Source Data
