# Supplementary material for: Candida albicans exploits N-acetylglucosamine as a gut signal to establish the balance between commensalism and pathogenesis
Source: Nat Commun. 2023 Jun 26;14:3796. doi: 10.1038/s41467-023-39284-w (PMC10293180; doi:10.1038/s41467-023-39284-w)
Supplement: Supplementary file 1 — Supplementary Information [file 41467_2023_39284_MOESM1_ESM.pdf]

**Supplementary Information:**

# *Candida albicans* exploits N-acetylglucosamine as a gut signal to establish the balance between commensalism and pathogenesis

Dandan Yang[1], Mao Zhang[2], Chang Su[2], Bin Dong[1], and Yang Lu[1*]

[1]Hubei Key Laboratory of Cell Homeostasis, College of Life Sciences, TaiKang Center for Life and Medical Sciences, Wuhan University, Wuhan 430072, China;

[2]Hubei Key Laboratory of Cell Homeostasis, College of Life Sciences, Wuhan University, Wuhan 430072, China.

[*]Correspondence: ylu7@whu.edu.cn

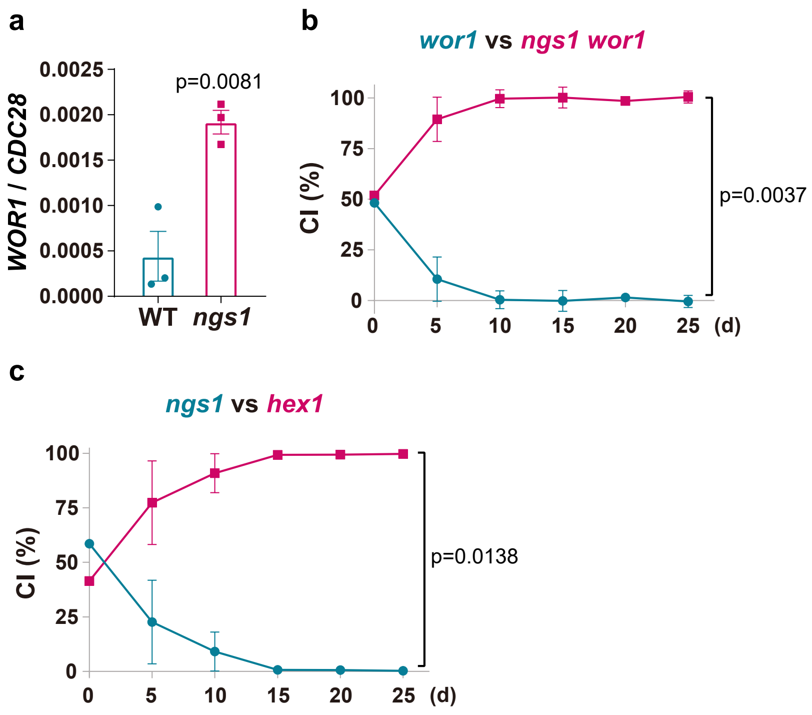


**Supplementary Fig. 1 The enhanced commensal fitness of *ngs1* mutant is not solely due to the increased *WOR1* expression.**

**a** The expression level of *WOR1* in wild-type strain and *ngs1* mutant recovered from contents of large intestines in mice at day 3 after oral gavage. The expression level was normalized with *CDC28*. Data are presented as the mean ± SEM. n=3 biologically independent samples. Significance was determined using the two-tailed unpaired Student's t test.

**b** Competition between *ngs1 wor1* double mutant and *wor1* single mutant.

**c** Competition between *ngs1* mutant and *hex1* mutant.

**b & c** n = 4 mice housed separately. Data are presented as the mean ± SEM. Significance was determined using the two-tailed paired Student's t test.

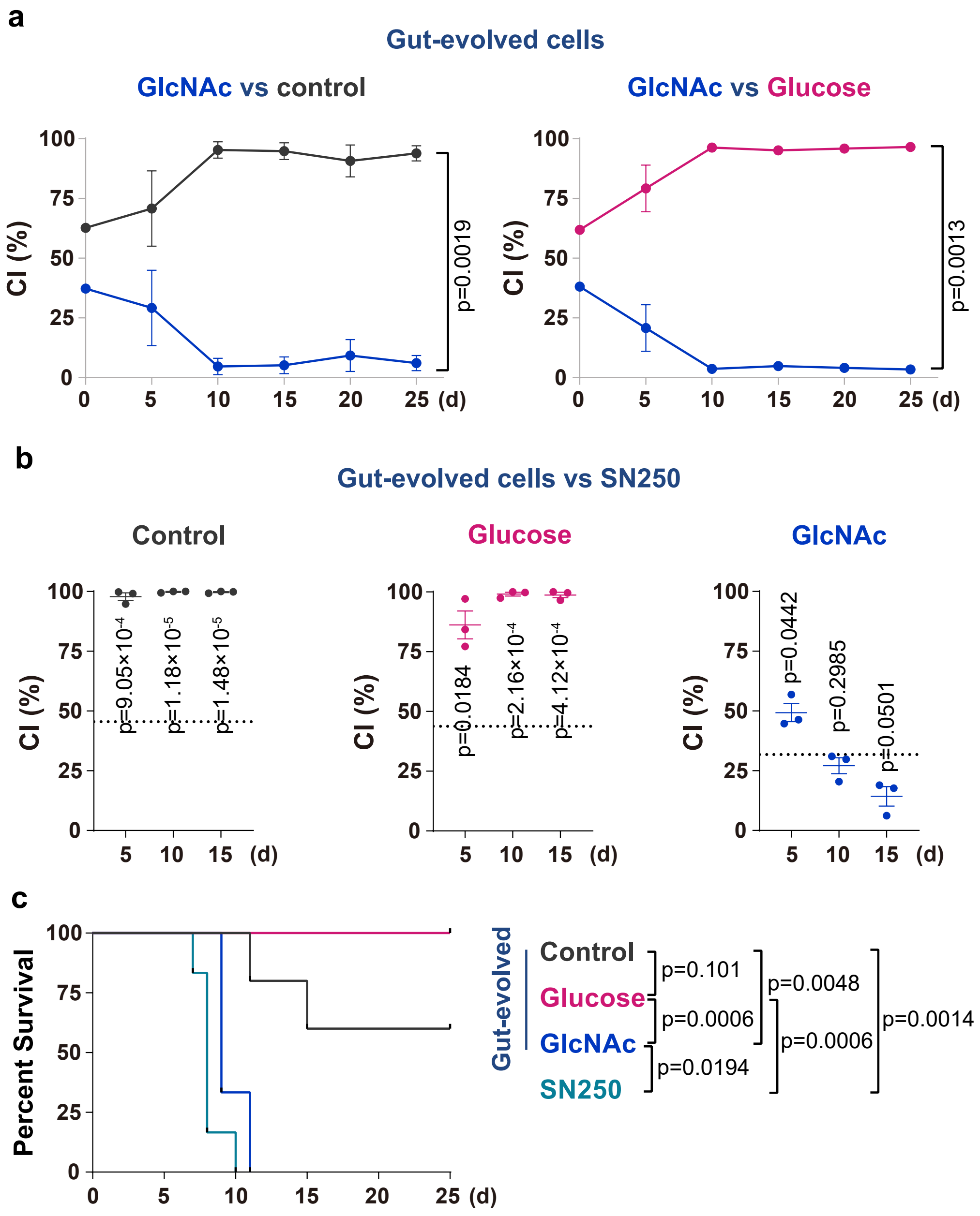


**Supplementary Fig. 2 GlcNAc attenuates competitive fitness of gut-evolved cells but retains their virulence potential.**

**a** Competition of gut-evolved cells between GlcNAc and control water (left), GlcNAc and glucose (right). Three clonal isolates from GlcNAc-evolved cells were randomly paired with that of control water or glucose for competitive fitness experiments. n = 3 mice housed separately. Data are presented as the mean ± SEM. Significance was determined using the two-tailed paired Student's t test.

**b** Competition between gut-evolved cells and original WT SN250. n = 3 mice housed separately. CI: the ratio of gut-evolved cells. Each data point indicates the CI of indicated strain in one animal. Dashed lines indicate the starting ratio. Data are

presented as the mean ± SEM. Significance was determined using the two-tailed unpaired Student's t test.

**c** The survival curve of mice injected with original WT (SN250) cells and cells harvested from mice treated with different sugars after 4 weekly serial passages. n = 6 mice. All recovered colonies was collected for the virulence assay. Significance was determined using the log-rank test.

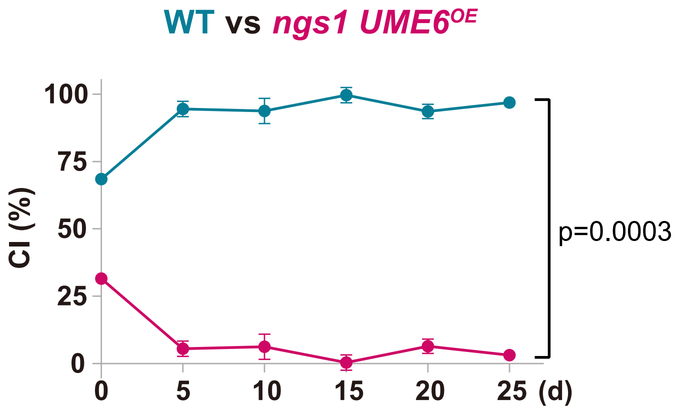


**Supplementary Fig. 3 Overexpression of *UME6* in *ngs1* produces a strong loss-of-fitness phenotype.** Competition between $ngs1^{UME6OE}$ and WT. n = 4 mice housed separately. Data are presented as the mean ± SEM. Significance was determined using the two-tailed paired Student's t test.

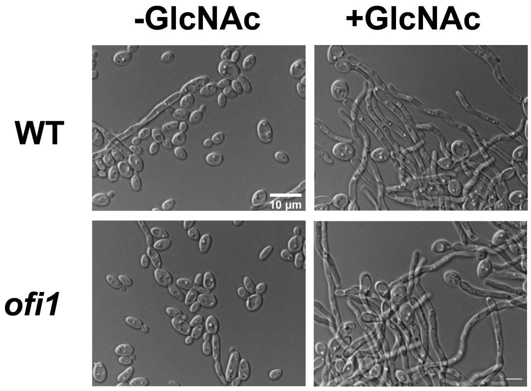


**Supplementary Fig.4 *ofi1* mutant displays no defect on hyphal growth in response to GlcNAc.** The hyphal induction was conducted as described in Fig. 4a. Representative images of three biologically independent experiments are shown. Scale bar, 10 μm.

## **Supplementary Table 1.** ***C. albicans*** **strains used in this study**

| Strain | Genotype | Reference |
|---|---|---|
| SN250 | *leu2Δ::C.m.LEU2/leu2Δ::C.d.HIS1, ura3Δ/URA3, his1Δ/his1Δ, arg4Δ/arg4Δ, iro1Δ/IRO1* | [1] |
| SC5314 | Wild type | [2] |
| BWP17 | *ura3Δ::imm434/ura3Δ::imm434, his1Δ::hisG/his1Δ::hisG, arg4Δ::hisG/arg4Δ::hisG* | [3] |
| *ngs1* | *his1Δ/his1Δ, leu2Δ/leu2Δ, arg4Δ/arg4Δ, URA3/ura3Δ::imm*$^{434}$*, IRO1/iro1Δ::imm*$^{434}$*, ngs1Δ::C.d.HIS1/ngs1Δ::C.m.LEU2* | [1] |
| *hex1* | *his1Δ/his1Δ, leu2Δ/leu2Δ, arg4Δ/arg4Δ, URA3/ura3Δ::imm*$^{434}$*, IRO1/iro1Δ::imm*$^{434}$*, hex1Δ::C.d.HIS1/hex1Δ::C.m.LEU2* | [1] |
| YLC41 | *leu2Δ::C.m.LEU2/leu2Δ::C.d.HIS1, ura3Δ/URA3, his1Δ/his1Δ, arg4Δ/arg4Δ, iro1Δ/IRO1, ADE2/ade2Δ::SAT1* | This study |
| YLC52 | *ura3Δ::imm*$^{434}$*/URA3, his1Δ::hisG/his1Δ::hisG, arg4Δ::hisG/ARG4, RP10/rp10Δ::HIS1* | This study |
| YLC53 | *ura3Δ::imm*$^{434}$*/URA3, his1Δ::hisG/his1Δ::hisG, arg4Δ::hisG/arg4Δ::hisG, ADE2/ade2Δ::SAT1, hxk1Δnag1Δdac1Δ::HIS1/ hxk1Δnag1Δdac1Δ::ARG4* | This study |
| YLC54 | *ura3Δ::imm*$^{434}$*/URA3, his1Δ::hisG/ HIS1, arg4Δ::hisG/ ARG4, wor1Δ/wor1Δ, Neut5L::FRT/Neut5L,* | This study |
| YLC55 | *his1Δ/his1Δ, leu2Δ/leu2Δ, ARG4/arg4Δ, URA3/ura3Δ::imm*$^{434}$*, IRO1/iro1Δ::imm*$^{434}$*, ngs1Δ::C.d.HIS1/ngs1Δ::C.m.LEU2, wor1Δ/wor1Δ, Neut5L::FRT/Neut5L, ADE2/ade2Δ::SAT1* | This study |
| UZ43 | *leu2Δ/leu2Δ, his1Δ/his1Δ, URA3/ura3Δ, IRO1/iro1Δ::imm*$^{434}$*, ume6Δ::CdHIS1/ume6Δ::CmLEU2* | [4] |
| HLY4427 | *his1Δ/his1Δ, leu2Δ/leu2Δ, arg4Δ/arg4Δ, ura3Δ/ura3Δ::imm*$^{434}$*, IRO1/iro1Δ::imm*$^{434}$*, ngs1Δ::C.d.HIS1/ngs1Δ::C.m.LEU2, hxk1Δ::URA3/hxk1Δ::ARG4* | [5] |
| YLC58 | *gig1Δ::FRT/gig1Δ::FRT* | This study |
| *ofi1* | *his1Δ/his1Δ, leu2Δ/leu2Δ, arg4Δ/arg4Δ, URA3/ura3Δ::imm*$^{434}$*, IRO1/iro1Δ::imm*$^{434}$*, ofi1Δ::C.d.HIS1/ofi1Δ::C.m.LEU2* | [1] |
| YLC60 | Wild type, *ADE2/ade2Δ::SAT1* | This study |
| YLC61 | *his1Δ/his1Δ, leu2Δ/leu2Δ, arg4Δ/arg4Δ, URA3/ura3Δ::imm*$^{434}$*, IRO1/iro1Δ::imm*$^{434}$*, ngs1Δ::C.d.HIS1/ngs1Δ::C.m.LEU2, ADE2/ade2Δ::SAT1* | This study |

## References


1 Noble, S. M., French, S., Kohn, L. A., Chen, V. & Johnson, A. D. Systematic screens of a Candida albicans homozygous deletion library decouple morphogenetic switching and pathogenicity. *Nat Genet* **42**, 590-U166, doi:10.1038/ng.605 (2010).

2 Fonzi, W. A. & Irwin, M. Y. Isogenic Strain Construction and Gene-Mapping in Candida-Albicans. *Genetics* **134**, 717-728 (1993).

3 Wilson, R. B., Davis, D. & Mitchell, A. P. Rapid hypothesis testing with Candida albicans through gene disruption with short homology regions. *J Bacteriol* **181**, 1868-1874, doi:Doi 10.1128/Jb.181.6.1868-1874.1999 (1999).

4 Zeidler, U. *et al.* UME6 is a crucial downstream target of other transcriptional regulators of true hyphal development in Candida albicans. *Fems Yeast Research* **9**, 126-142, doi:10.1111/j.1567-1364.2008.00459.x (2009).

5 Su, C., Lu, Y. & Liu, H. P. N-acetylglucosamine sensing by a GCN5-related N-acetyltransferase induces transcription via chromatin histone acetylation in fungi. *Nat Commun* **7**, doi:ARTN 12916
10.1038/ncomms12916 (2016).
