# Supplementary material for: Candida albicans exploits N-acetylglucosamine as a gut signal to establish the balance between commensalism and pathogenesis
Source: Nat Commun. 2023 Jun 26;14:3796. doi: 10.1038/s41467-023-39284-w (PMC10293180; doi:10.1038/s41467-023-39284-w)
Supplement: Supplementary file 2 — Description of Additional Supplementary Files [file 41467_2023_39284_MOESM2_ESM.docx]

**Description of Additional Supplementary Files**

File Name: Supplementary Data 1

Description: Non-synonymous variations between GlcNAc-evolved strains and strains evolved in control water in verified open reading frames (ORFs).

File Name: Supplementary Data 2

Description: mRNA-Seq data for wild-type *C. albicans* and *ngs1* propagated under in vitro GlcNAc and glucose conditions and in a mouse model of GI colonization. Differentially expressed genes were defined by fold change ≥1.5 and a P value of < 0.05 found by DESeq2 (1.34.0 in Rstudio). No adjustment for multiple comparisons was made.

File Name: Supplementary Data 3

Description: Primers used in this study.
