# Supplementary material for: Candida albicans exploits N-acetylglucosamine as a gut signal to establish the balance between commensalism and pathogenesis
Source: Nat Commun. 2023 Jun 26;14:3796. doi: 10.1038/s41467-023-39284-w (PMC10293180; doi:10.1038/s41467-023-39284-w)
Supplement: Supplementary file 6 — Reporting Summary [file 41467_2023_39284_MOESM6_ESM.pdf]

nature portfolio

Corresponding author(s): Yang Lu

Last updated by author(s): May 28, 2023

Reporting Summary

Nature Portfolio wishes to improve the reproducibility of the work that we publish. This form provides structure for consistency and transparency in reporting. For further information on Nature Portfolio policies, see our Editorial Policies and the Editorial Policy Checklist.

Statistics

For all statistical analyses, confirm that the following items are present in the figure legend, table legend, main text, or Methods section.

n/a	Confirmed	
☐	☒	The exact sample size (*n*) for each experimental group/condition, given as a discrete number and unit of measurement
☐	☒	A statement on whether measurements were taken from distinct samples or whether the same sample was measured repeatedly
☐	☒	The statistical test(s) used AND whether they are one- or two-sided<br>*Only common tests should be described solely by name; describe more complex techniques in the Methods section.*
☒	☐	A description of all covariates tested
☐	☒	A description of any assumptions or corrections, such as tests of normality and adjustment for multiple comparisons
☐	☒	A full description of the statistical parameters including central tendency (e.g. means) or other basic estimates (e.g. regression coefficient) AND variation (e.g. standard deviation) or associated estimates of uncertainty (e.g. confidence intervals)
☐	☒	For null hypothesis testing, the test statistic (e.g. *F*, *t*, *r*) with confidence intervals, effect sizes, degrees of freedom and *P* value noted<br>*Give P values as exact values whenever suitable.*
☒	☐	For Bayesian analysis, information on the choice of priors and Markov chain Monte Carlo settings
☒	☐	For hierarchical and complex designs, identification of the appropriate level for tests and full reporting of outcomes
☒	☐	Estimates of effect sizes (e.g. Cohen's *d*, Pearson's *r*), indicating how they were calculated

Our web collection on statistics for biologists contains articles on many of the points above.

Software and code

Policy information about availability of computer code

Data collection	ChemiDoc MP imaging system (Bio-Rad) was used to capture colony images. CFX connect Real-Time system from Bio-Rad was used to acquire qPCR data. Micrographs for cell morphology were taken on a Leica 2500 microscope.
Data analysis	Graphpad Prism 9.0; ggplot2 (3.4.1); DEseq2 (1.34.0 in Rstudio); Fastp; BWA (0.7.17); SAMTOOLS; Genome Analysis ToolKit (GATK, v4.1.2); DELLY (0.8.1); CNVnator (0.4); snpEff (4.3t).

For manuscripts utilizing custom algorithms or software that are central to the research but not yet described in published literature, software must be made available to editors and reviewers. We strongly encourage code deposition in a community repository (e.g. GitHub). See the Nature Portfolio guidelines for submitting code & software for further information.

Data

Policy information about availability of data

All manuscripts must include a data availability statement. This statement should provide the following information, where applicable:

- Accession codes, unique identifiers, or web links for publicly available datasets
- A description of any restrictions on data availability
- For clinical datasets or third party data, please ensure that the statement adheres to our policy

RNA-Seq data and genome sequencing data that support the findings of this study have been deposited in the Genome Sequence Archive (GSA) under the accession code CRA010506 [https://bigd.big.ac.cn/gsa/browse/CRA010506] and CRA010507 [https://bigd.big.ac.cn/gsa/browse/CRA010507] respectively. Reference genomes and genome annotations were obtained from Candida Genome Database. The generated strains in this study are available from the corresponding author

upon request. Source data are provided with this paper.

Human research participants

Policy information about studies involving human research participants and Sex and Gender in Research.

Reporting on sex and gender	n/a
Population characteristics	n/a
Recruitment	n/a
Ethics oversight	n/a

Note that full information on the approval of the study protocol must also be provided in the manuscript.

Field-specific reporting

Please select the one below that is the best fit for your research. If you are not sure, read the appropriate sections before making your selection.

[x] Life sciences [ ] Behavioural & social sciences [ ] Ecological, evolutionary & environmental sciences

For a reference copy of the document with all sections, see nature.com/documents/nr-reporting-summary-flat.pdf

Life sciences study design

All studies must disclose on these points even when the disclosure is negative.

Sample size	No sample-size calculation was performed. Sample size was chosen according to the experiment type and based on what is standard practice in the field (reference 7) and is mentioned for each experiment.
Data exclusions	No data were excluded.
Replication	All experiments were performed with at least three biological repeats except indicated in the figure legends, and all attempts at replication were successful.
Randomization	Sample allocation was random in all experiments.
Blinding	Blinding of the experimenters to group allocation was not implemented during data acquisition and analysis. To minimal potential bias, control and experimental groups were tested under the same experimental conditions when necessary, and data from all groups were analyzed using identical criteria and methods.

Reporting for specific materials, systems and methods

We require information from authors about some types of materials, experimental systems and methods used in many studies. Here, indicate whether each material, system or method listed is relevant to your study. If you are not sure if a list item applies to your research, read the appropriate section before selecting a response.

Materials & experimental systems

n/a	Involved in the study	
[x]	[ ]	Antibodies
[x]	[ ]	Eukaryotic cell lines
[x]	[ ]	Palaeontology and archaeology
[ ]	[x]	Animals and other organisms
[x]	[ ]	Clinical data
[x]	[ ]	Dual use research of concern

Methods

n/a	Involved in the study	
[x]	[ ]	ChIP-seq
[x]	[ ]	Flow cytometry
[x]	[ ]	MRI-based neuroimaging

Animals and other research organisms

Policy information about studies involving animals; ARRIVE guidelines recommended for reporting animal research, and Sex and Gender in Research

Laboratory animals	6-week-old male ICR mice; 6-8-week-old BABL/c male/female mice
Wild animals	No wild animals were used in this study.
Reporting on sex	In the present study, we did not examined sex differences. Male mice were used for virulence-related experiments, while female mice were used for experiments regarding commensal fitness examination, based on what is standard practice in the field (reference 6 and 7).
Field-collected samples	No field-collected samples were used in this study.
Ethics oversight	All animal experiments were approved by the Institutional Animal Care and Use Committee (IACUC) at Wuhan University and performed as outlined in the guide for the care and use of laboratory animals issued by the Ministry of Science and Technology of the People's Republic of China.

Note that full information on the approval of the study protocol must also be provided in the manuscript.
